# P-573. Investigation of Institutional Protocols During the 2022 Mpox Outbreak: A SHEA Research Network Survey

**DOI:** 10.1093/ofid/ofaf695.788

**Published:** 2026-01-11

**Authors:** Jordan Beam, Elliot Herron, Rachael Cowan, Nicholas Van Wagoner, Rachael A Lee

**Affiliations:** University of Alabama at Birmingham Heersink School of Medicine, Hoover, AL; University of Alabama Birmingham Heersink School of Medicine, Birmingham, Alabama; University of Alabama Birmingham Heersink School of Medicine, Birmingham, Alabama; University of Alabama at Birmingham, Birmingham, Alabama; University of Alabama at Birmingham, Birmingham, Alabama

## Abstract

**Background:**

The 2022 Mpox outbreak affected many non-endemic countries worldwide and disproportionately impacted men who have sex with men (MSM). By May 2023, over 80,000 cases and 140 deaths were reported from 111 countries and territories. The unprecedented human-to-human transmission highlighted the need to assess system responses. This study evaluates institutional handling of Mpox through a survey of individuals responsible for outbreak protocols.

**Figure:**
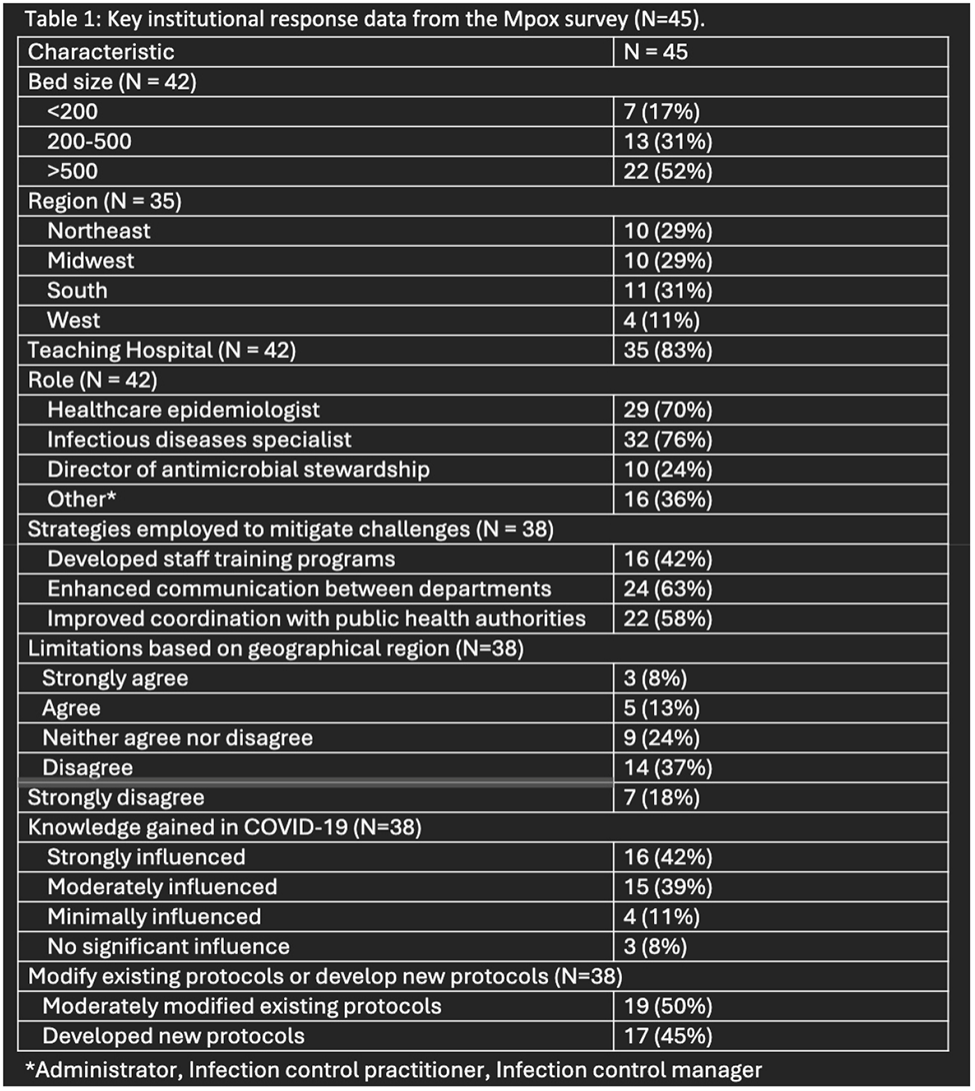

**Figure:**
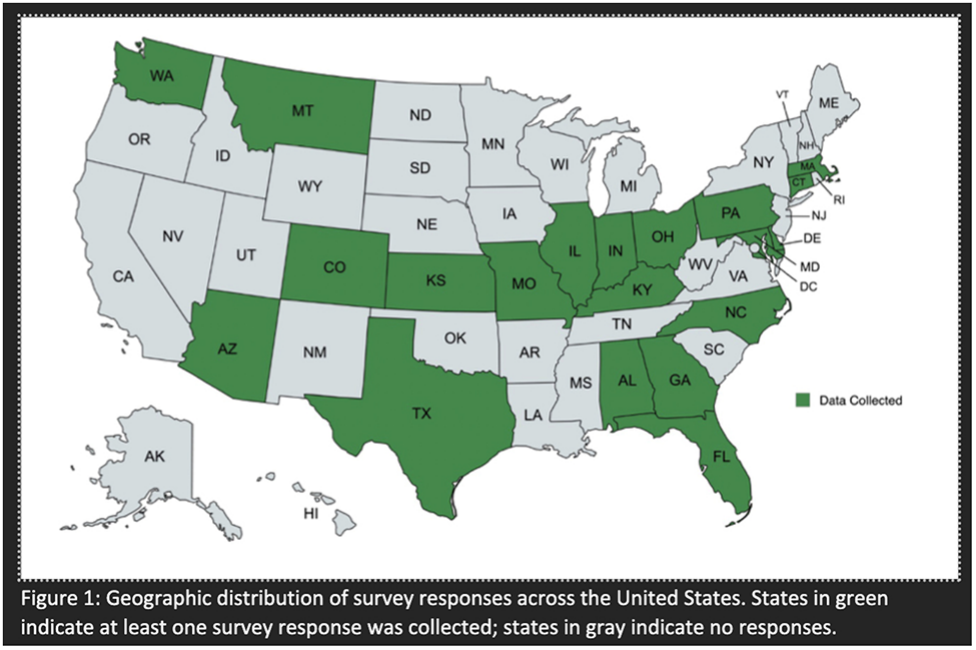

**Methods:**

A cross-sectional survey was conducted between December 2024 and April 2025 among infectious disease specialists across America. Institutions were recruited through the Society for Healthcare Epidemiology of America (SHEA) Research Network. Descriptive statistics were used to summarize the data.

**Figure:**
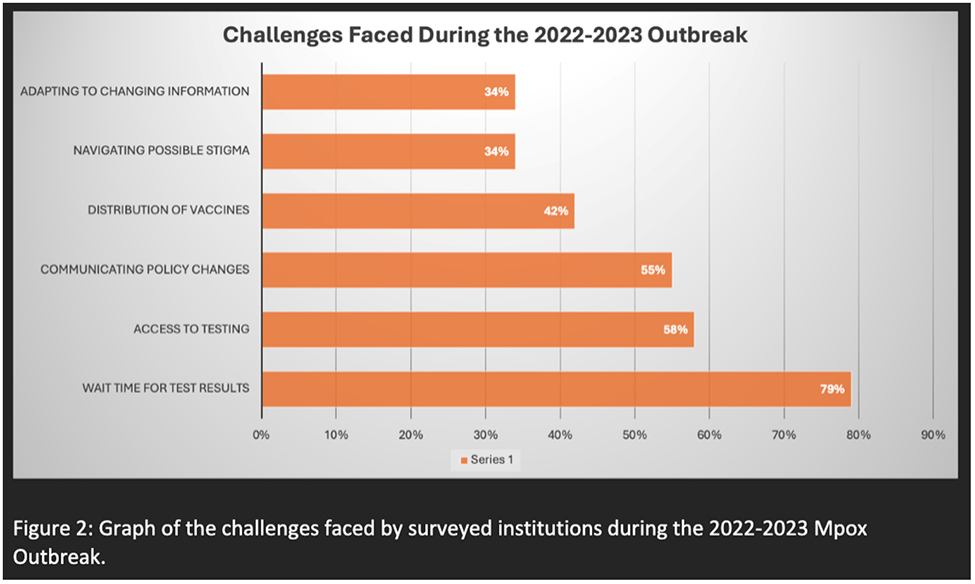

**Results:**

Key results are reported in Table 1. The cohort included 45 institutions across 20 states representing various roles and institution types (Figure 1). Main challenges included delays in test results, access to supplies, and communicating policy changes (Figure 2). To mitigate these challenges, institutions enhanced communication, coordination, and staff training programs. Only 21% reported resources limitations based on geographical region. COVID-19 experiences moderately to strongly influenced 81% of institutions’ responses to the outbreak, and most institutions either moderately modified existing protocols or developed new ones.

**Conclusion:**

These reported findings highlight the variability in institutional response to the 2022 Mpox outbreak across the US and emphasize the challenges related to resource allocation and timely communication during public health emergencies. Understanding the experiences of those directly involved in protocol implementation provides valuable insight into systemic strengths and gaps, offering guidance for more coordinated and efficient responses in future outbreaks.

**Disclosures:**

All Authors: No reported disclosures

